# MiR-4435 is an UQCRB-related circulating miRNA in human colorectal cancer

**DOI:** 10.1038/s41598-020-59610-2

**Published:** 2020-02-18

**Authors:** Ji Won Hong, Jung Min Kim, Jeong Eun Kim, Hee Cho, Dasol Kim, Wankyu Kim, Jong-Won Oh, Ho Jeong Kwon

**Affiliations:** 1 Department of Biotechnology, College of Life Science and Biotechnology, Yonsei University, Seoul, 03722 Republic of Korea; 2 Ewha Research Center for Systems Biology, Division of Molecular & Life Sciences, Ewha Womans University, Seoul, Republic of Korea; 3 Department of Internal Medicine, Yonsei University College of Medicine, Seoul, 03722 Republic of Korea

**Keywords:** Colon cancer, Diagnostic markers

## Abstract

Ubiquinol-cytochrome c reductase (UQCRB), a subunit of the mitochondrial complex III, is highly expressed in tissues from colorectal cancer patients. Since UQCRB is highly expressed in colorectal cancer, we investigated miRNAs from mutant UQCRB-expressing cell lines to identify new miRNA biomarkers. After sequencing miRNAs in the mutant UQCRB-expressing cell lines, miR-4435 was selected as a potential biomarker candidate from the six up-regulated miRNAs. The expression level of miR-4435 in the mutant UQCRB-expressing cell lines and colon cancer was increased. Notably, the expression level of miR-4435 was increased in exosomes isolated from cell culture medium, suggesting that miR-4435 is closely related to colon cancer and that large amounts of miR-4435 may be secreted outside of the cells through exosomes. Additionally, exosomes extracted from the serum samples of colorectal cancer patients showed increased miR-4435 levels depending on the cancer progression stage. Moreover, analyses of a miRNA database and mRNA-sequencing data of the mutant UQCRB-expressing cell lines revealed that *TIMP3*, a tumor suppressor, could be a target of miR-4435. Additionally, the expression of miR-4435 was suppressed by UQCRB inhibitor treatment whereas TIMP3 was up-regulated. Upregulation of TIMP3 decreased proliferation of the mutant UQCRB-expressing cell lines and a colorectal cancer cell line. TIMP3 was also upregulated in response to miR-4435 inhibitor and UQCRB inhibitor treatments. Furthermore, these findings suggest that miR-4435 is related to an oncogenic function in UQCRB related disease, CRC, and that effects migration and invasion on mutant UQCRB-expressing cell lines and colorectal cancer cell. In conclusion, our results identified miR-4435 as a potential circulating miRNA biomarker of colorectal cancer associated with UQCRB.

## Introduction

Mitochondria play a crucial role in energy conversion to produce ATP and contribute to various biosynthetic steps. The electron transport complex (ETC) of the mitochondria generates an electrochemical proton gradient to produce energy by ATP synthesis^1^. Mitochondrial dysfunctions cause many mitochondrial diseases and are related to many diseases such as metabolic disease and cancer^1–5^.

Ubiquinol-cytochrome c reductase binding protein (UQCRB) is localized in the mitochondrial complex III where it plays an important role in electron transport and maintenance of complex III^6^. Additionally, UQCRB is involved in the generation of mitochonrial reactive oxygen species (mROS)- and hypoxia-inducible factor (HIF)-mediated angiogenesis and regulates vascular endothelial growth factor receptor 2 (VEGFR2) signaling-induced angiogenesis^7,8^. It is also known that UQCRB is a target protein of terpestacin, an anti-angiogenic natural small molecule. Notably, it has been shown that terpestacin binds UQCRB and has anti-angiogenic activity^9^. In addition, several reports have revealed UQCRB plays a role in cancer by identifying genetic variations of UQCRB in hepatocellular carcinoma^10^, ovarian cancer^11^, pancreatic ductal adenocarcinoma^12^, and colorectal cancer (CRC)^13^.

Mutations in UQCRB can result in mitochondrial defects and associated disorders. Previously, a case study of metabolic disorders such as hypoglycemia and lactic acidosis occurred in a Turkish girl patient with frame shift mutation in the *UQCRB* gene was reported^14^. A girl with a mutation in the UQCRB gene showed hypoglycemia and lactic acidosis during a metabolic crisis as a baby. Based on this, we constructed a mutant UQCRB expressing clone as the same as human mutation of *UQCRB* and obtained two stable cell lines expressing the mutant UQCRB with different expression levels. MT1 is a cell line expressing higher mutant UQCRB level whereas MT2 is a moderate level of mutant UQCRB. We used these stable cell lines to determine the biological functions of UQCRB in angiogenesis. The mutant UQCRB-expressing cell lines showed remarkably increased cell growth and pro-angiogenesis activities. In addition, the mitochondria of the mutant UQCRB-expressing cell lines had morphological abnormalities and were more sensitive to UQCRB inhibitors^15^. MicroRNAs (miRNAs) are small non-coding RNAs that are 21–23 nucleotides in length. Mature miRNAs bind target mRNAs at complementary sites in the 3′ untranslated regions (UTRs) of the latter, which results in mRNA silencing. The post-transcriptional regulation of miRNAs has been reported^16,17^. In addition, miRNAs can act as tumor suppressors or oncogenes^18^. Hence, miRNAs are important for the control of numerous physiological and pathological processes, such as cell proliferation, invasion and migration, metastasis, and others^19,20^. Although there are few studies on the relationship between miRNAs and UQCRB, we recently reported that hsa-miR-10a-5p is associated with UQCRB. The downregulation of miR-10a-5p activates the cholesterol pathway in mutant UQCRB-expressing cells by targeting the cholesterol-synthesizing enzyme, suggesting a possible role of miRNA related with UQCRB in the proliferation of cancer cells^21^. In addition, these miRNAs are stable in serum and plasma and their expression levels vary under different disease conditions, such as cancer^22^. Many reports have suggested using miRNA expression profiles as a biomarker of various diseases. A recent paper showed that serum miR-155 was upregulated in CRC patients compared with that in healthy controls, suggesting serum miR-155 could be used as a CRC biomarker^23^. In addition, Lv, Z. C. *et al*. reported four miRNAs associated with overall survival for non-small-cell lung cancer (NSCLC)^24^. However, the mechanism and regulation of these miRNAs in disease is still not clear.

CRC is the third most common type of cancer worldwide and a leading cause of death. Currently, surgery is the main treatment in the early stage of cancer. However, patients are often not diagnosed until they are at an advanced stage of disease, sometimes with distant metastases already present^25^. Recently, the relationship between UQCRB and CRC has been studied. UQCRB gene and protein levels were highly expressed in tissues of colon cancer patients compared with that in adjacent non-tumor tissues^26^. This implies UQCRB is possibly oncogenic in CRC and can be used as a possible biomarker for diagnosis. In this study, we investigated miRNAs from mutant UQCRB-expressing cell lines to identify new miRNA biomarkers in respect with CRC. In addition, we performed miRNA analysis of human CRC patient serum samples to explore the clinical impact of UQCRB and identify miR-4435 as a potential circulating miRNA biomarker of UQCRB-related cancer of CRC.

## Results

### Selected miRNAs were upregulated in mutant UQCRB-expressing cell lines and CRC cells

**Figure 1:**
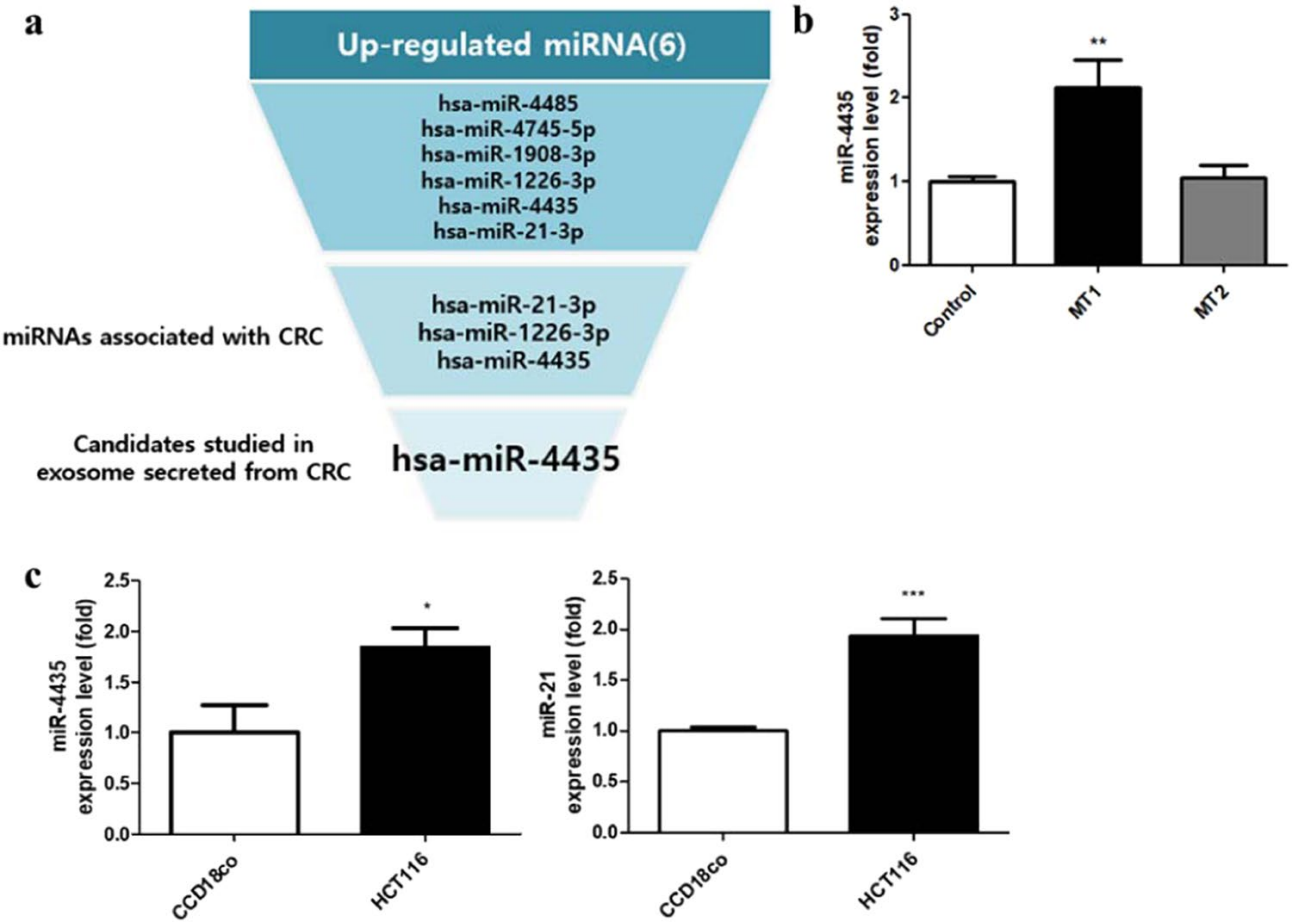
Selected miRNAs were upregulated in mutant UQCRB-expressing cells and colon cancer cells. (**a**) Schematic of selection process for miRNAs associated with mutant UQCRB-expressing cells and colorectal cancer. FC represents the differential expression between control and MT cells. CPM represents the normalized expression of each miRNA. (**b**) Validation of the selected miRNAs using qRT-PCR in mutant UQCRB-expressing cell lines. (**c**) Validation of selected miRNAs using qRT-PCR in colon cancer cells. All quantified data are presented as mean ± standard error (±S.E.M) compared to control (*p < 0.05, **p < 0.01, ***p < 0.0001).

We previously performed miRNA-sequencing in HEK293 and mutant UQCRB-expressing HEK293 cell lines (MT1 and MT2) to analyze differentially expressed miRNAs in mutant cells compared to control cells (HEK293)^21^. Firstly, 1,338 and 1,195 differentially expressed miRNAs were identified in the MT1 and MT2 cell lines, respectively, compared to control. Of these miRNAs, six miRNAs (hsa-miR-4485, -4745-5p, -1908-3p, -1226-3p, -4435, -21-3p) were considered significant and selected as candidates considering three criteria: |log_2_FC| > 1, |log_2_CPM| > 2, FDR < 0.05 compared to control. A schematic for the miRNA candidate selection procedure is shown in Fig. 1a. To begin with, miRNAs associated with CRC, a disease associated with UQCRB, were selected, leaving three candidates (miR-21-3p, miR-1226-3p, miR-4435), which were already known to be increased in CRC via screening^27–29^. Among these three candidates, miR-4435 was selected as a final candidate because it has been shown to be secreted via exosome by CRC cells^30^. Next, we validated whether miR-4435 level was upregulated in mutant UQCRB-expressing cell lines compared to the control cell line (HEK293) by quantitative RT-PCR. Confirming the miRNA-sequencing data, miR-4435 levels were upregulated in mutant UQCRB-expressing cell lines compared to the control cell line (HEK293) (Fig. 1b). Levels of a known miRNA CRC biomarker, miR-21, is also increased in CRC^31^, and was used as a positive control. Expression levels of both miR-21 and miR-4435 were increased two-fold in the CRC cell line, HCT116, when compared to the normal colon cell line (Fig. 1c). Additionally, this candidate miRNA is specifically expressed in the colorectal cancer. We examined the expression level of miR-4435 in gastric cancer (YCC16), liver cancer (HepG2), and lung cancer (H1299), including colon cancer (HCT116), which are known to be highly prevalent. The result exhibited that the miR-4435 is specifically expressed in colorectal cancers. The second highest expression level was shown in gastric cancer which is expected due to the influence of gastrointestinal tract (Supplementary Fig. 1). Together, these results demonstrated that miR-4435 was up-regulated in mutant UQCRB-expressing cell lines and colon cancer cells.

### Selected-miRNAs were up-regulated in exosomes

**Figure 2:**
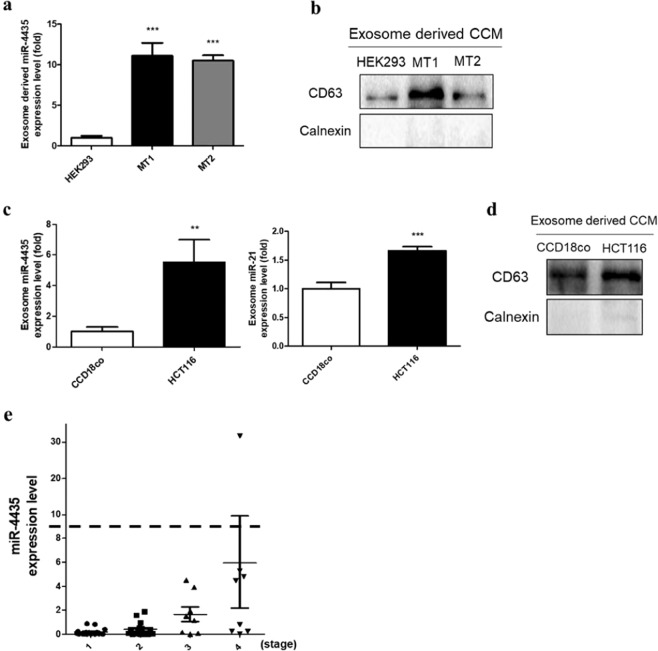
MiR-4435 was secreted by exosomes. (**a**) Expression levels of miR-4435 in exosomes secreted from mutant UQCRB-expressing cell lines were analyzed by qRT-PCR. (**b**) Detection of exosome markers in exosomes from mutant UQCRB-expressing cell cultured media. CD63 as a positive marker of exosomes. Calnexin as a negative marker of exosomes. (**c**) The expression levels of miR-4435 (left) and miR-21 (right) in exosomes secreted from colon cancer cells were analyzed by qRT-PCR. (**d**) Detection of exosome markers in exosomes from colon cancer cells cultured media. (**e**) Validation of miR-4435 in serum of colorectal cancer patients using qRT-PCR. The expression level of each patient. All quantified data are presented as mean ± S.E.M compared to control (*p < 0.05, **p < 0.01, ***p < 0.0001). These all images are representative of 3 repeated independently repeated experiments.

Our results showed that selected miRNA, miR-4435, was upregulated in mutant UQCRB-expressing and CRC cells. We then examined whether miR-4435 is secreted via exosomes. The mutant UQCRB-expressing cells and CRC cells were incubated for 72 h in culture media. The exosomes were isolated from the cell culture media (CCM) and expression levels of the candidate miRNAs in the exosomes were measured. The expression level of miR-4435 in the isolated exosomes was higher in the mutant UQCRB-expressing cell lines than in the control (HEK293) (Fig. 2a). To validate that we isolated exosomes and performed western blotting for exosome-specific markers (CD63 and calnexin). The result demonstrated that exosomes were successfully isolated from the CCM (Fig. 2b). In parallel, we measured the levels of miR-4435 secreted via exosome in CRC cells.

Compared with normal colon cells, the expression level of colorectal cancer cells was higher in miR-4435 than miR-21 (Fig. 2c). Again, western blotting for exosome-specific markers showed that the exosomes were properly isolated (Fig. 2d). Taken together, our results revealed that miR-4435 was highly expressed in the mutant UQCRB-expressing cell lines and CRC cells, and exosomes secreted from these cell lines also have higher levels of miR-4435.

Next, we examined the expression of miR-4435 in serum samples from CRC patients. Exosomes were isolated from serum samples of CRC patients and the miR-4435 expression levels in the exosomes were measured by qRT-PCR. The serum samples of patients of various ages with CRC from stage 1 to stage 4 were analyzed (Table 1). Levels of miR-4435 were correlated to the stage of CRC where miR-4435 expression increased as CRC stage advanced (Fig. 2e). Meanwhile, the expression levels of miR-21 in the serum samples of patients did not show any correlation to the stage (Supplementary Fig. 2). Collectively, these results demonstrated the possibility of miR-4435 as a circulating miRNA biomarker for diagnosing specific stages of CRC. Notably, miR-4435 was expected to be closely related to the progression of CRC.

**Table 1:** Clinical characteristics of 48 CRC patients at the time of diagnosis.

Parameter	Number of patients	% of patients	Range
Gender			
Male	24	50	
Female	24	50	
Age at diagnosis (year)			
<65	28	58	29–64
≥65	20	42	65–81
Clinical stage			
1	16	33	
2	16	33	
3	8	17	
4	8	17	

Abbreviation: CRC, Colorectal cancer.

### UQCRB-related key miRNA targeted tumor suppressor gene *TIMP*

**Figure 3:**
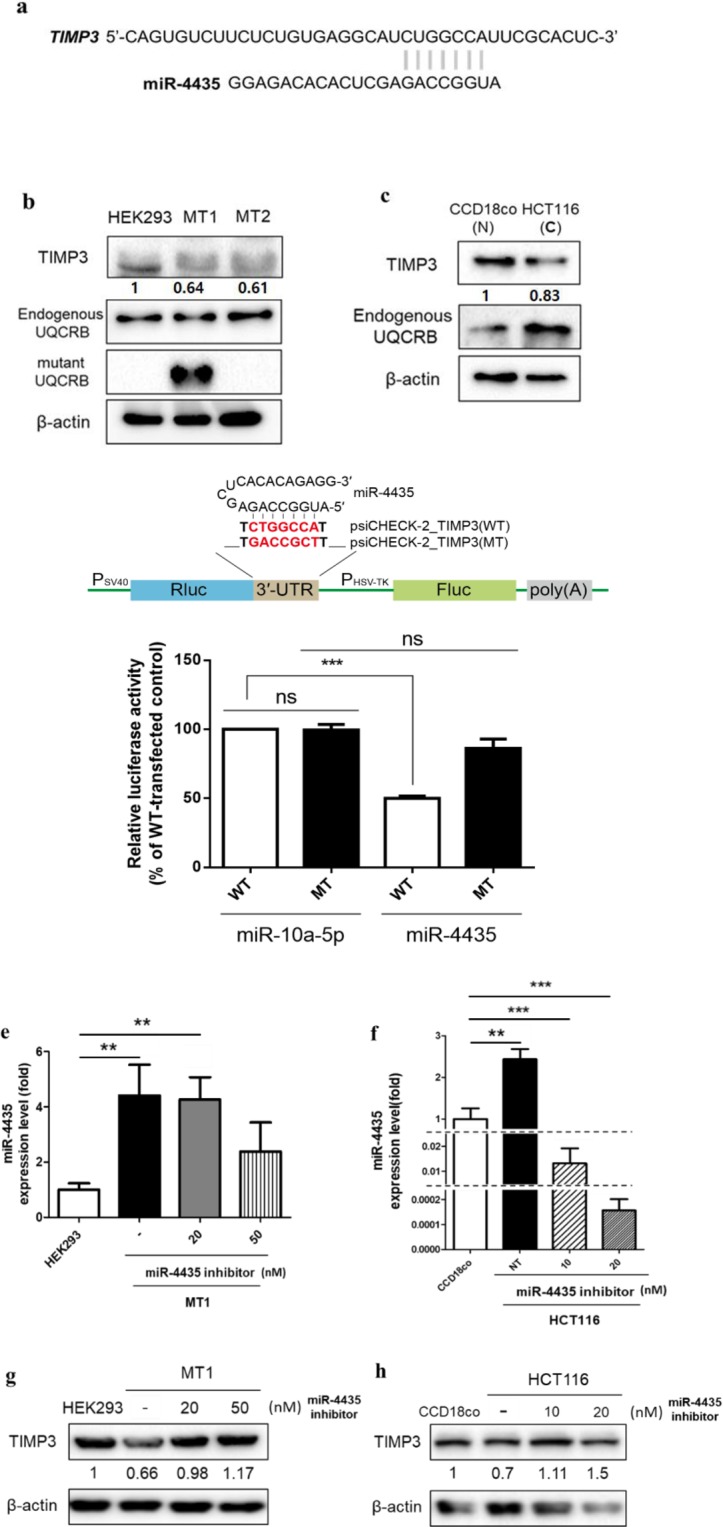
*TIMP3*, a tumor suppressor gene, as a potential target of MiR-4435. (**a**) miR-4435 and *TIMP3* seed sequences identified using miRmap database. (**b**,**c**) Protein expression levels of TIMP3 in mutant UQCRB-expressing cell lines and colon cancer cells. β-actin was used as an internal control. (**d**) Schematic representation of reporter plasmids carrying a miR-4435 target site [psiCHECK-2_TIMP3(WT)] or its mismatched target [psiCHECK-2_TIMP3(MT)] at their 3′-UTR (top). The miR-4435 core seed-matched sequence and its mutated one were shown in red. HEK293 cells were co-transfected with each indicated reporter plasmid and miR-10a-5p mimic or miR-4435 mimic. At 24 h post-transfection, normalized luciferase activity (Rluc/Fluc) compared with WT vector-transfected control was determined (bottom). miR-10a-5p, a miRNA mimic used as a control. All quantified data are presented as mean ± S.E.M compared to control. (***indicates *p* < 0.001). (**e**,**f**) Expression levels of miR-4435 after transfection of mutant UQCRB-expressing cell lines and colon cancer cells with miR-4435 inhibitor. (**g,h**) Expression levels of TIMP3 after transfection of mutant UQCRB-expressing cell lines and colon cancer cells with miR-4435 inhibitor. β-actin was used as an internal control. All quantified data are presented as mean ± S.E.M compared to control (*p < 0.05, **p < 0.01, ***p < 0.0001). These all images are representative of 3 repeated independently repeated experiments.

To determine what gene miR-4435 targets, we compared mRNA-sequencing results from mutant UQCRB-expressing cell lines (MT1 and MT2) to control. Among these mRNAs, 133 mRNAs were selected as significantly down-regulated candidates considering |log2(FC)| > 2 compared to control. We used the target prediction program, miRDB, to identify target mRNA of miR-4435. The expected miR-4435 target genes were 474 in total. We compared our mRNA-sequencing results in mutant UQCRB-expressing cell lines and the miRDB results to find common genes. Among the common genes, *TIMP3* was elucidated as a target gene candidate with high target score (53). The seed sequences of miR-4435 and *TIMP3* are shown in Fig. 3a. *TIMP3* has low expression level in CRC and is known as a tumor suppressor gene^32^. Thus, we examined the protein level of TIMP3 and revealed that the expression level was lower in the mutant UQCRB-expressing cell line than that in HEK293 (Fig. 3b). Furthermore, endogenous UQCRB levels in CRC cells were higher than those in normal control cells, CCD18Co. Conversely, protein level of TIMP3, the predicted target of miR-4435, was lower than controls (Fig. 3c). To validate whether TIMP3 was a direct target of miR-4435, we performed a dual luciferase reporter assay using a psiCHECK-2 vector that contains TIMP3 3′-UTR segment with wild-type or mutated miRNA seed sequence (Fig. 3d, top). As expected, the relative luciferase activities (Rluc/Fluc) were significantly reduced by miR-4435 (50 ± 1% of control), whereas miR-10a-5p mimic had no effect in cells transfected with the wild-type reporter (WT). This inhibition was completely abolished when the miR-4435 binding site was mutated. These results demonstrate that *TIMP3* is the direct target of miR-4435. In addition, miR-4435 inhibitor was transfected into mutant UQCRB-expressing cell lines and colon cancer cells to validate *TIMP3* as a target of miR-4435 further. In mutant UQCRB-expressing cell lines transfected with 50 nM miR-4435 inhibitor, we measured expression levels of miR-4435 by qRT-PCR and determined levels of miR-4435 were decreased about 50% (Fig. 3d). In CRC cells, the expression levels of miR-4435 were inhibited in a concentration-dependent manner by miR-4435 inhibitor treatment (Fig. 3e). Next, we examined the protein level of TIMP3 after down-regulation of miR-4435. Protein levels of TIMP3 were increased in mutant UQCRB-expressing cells transfected with miR-4435 inhibitor (Fig. 3f). In the colon cancer cells, the protein level of TIMP3 was also increased with the miR-4435 inhibitor treatment compared to non-treatment (Fig. 3g). These results demonstrated that miR-4435 targets *TIMP3*, and miR-4435 regulates the relationship between UQCRB and *TIMP3*, which ultimately contributes to tumorigenesis of CRC in part.

### MiR-4435 is regulated by the UQCRB inhibitor, A1938

**Figure 4:**
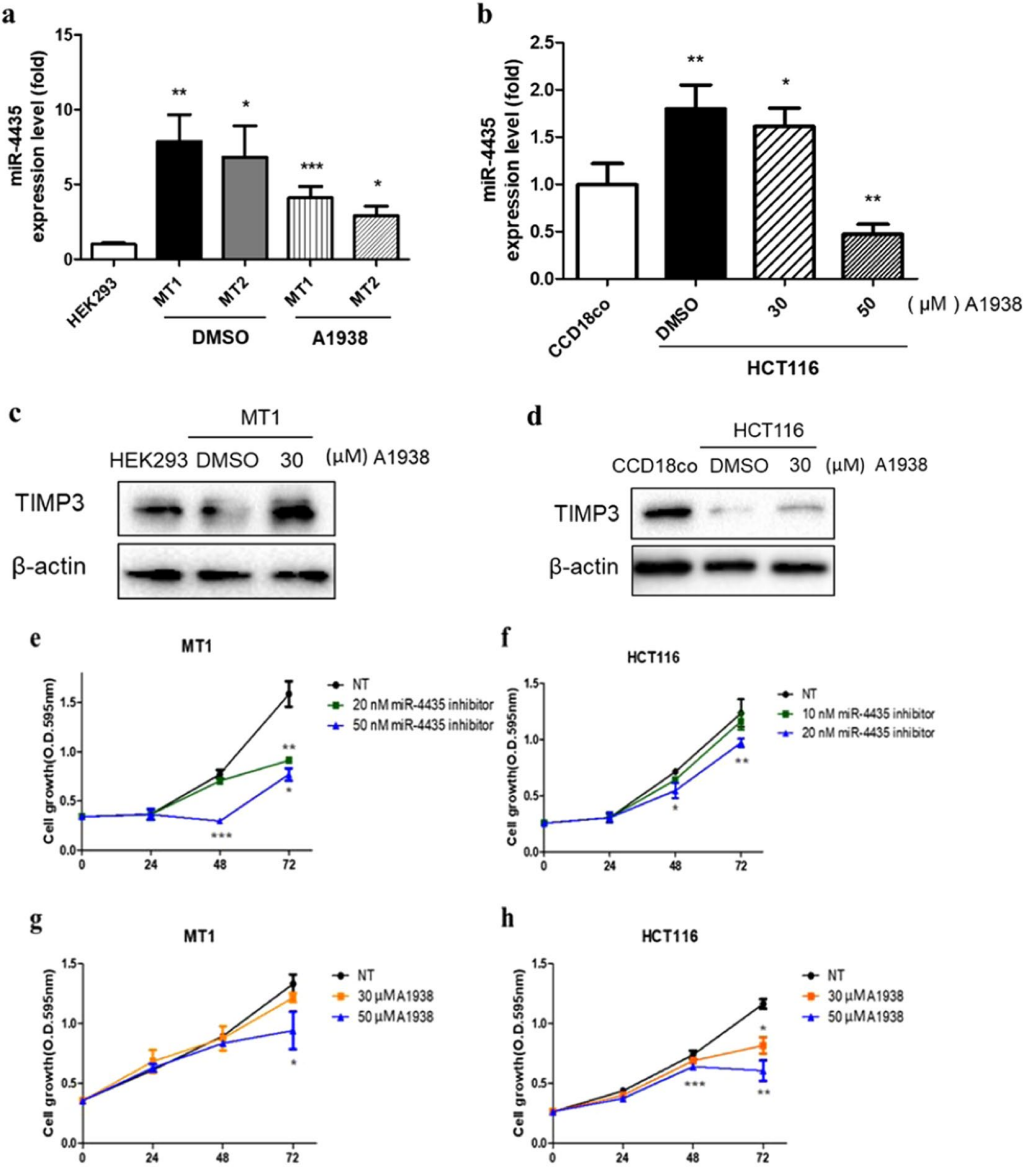
MiR-4435 is regulated by the UQCRB inhibitor, A1938. (**a**,**b**) Effect of A1938 on miR-4435 expression levels in mutant UQCRB-expressing cell lines and colon cancer cells. (**c**,**d**) Effect of A1938 on TIMP3 expression levels in mutant UQCRB-expressing cell lines and colon cancer cells. β-actin was used as an internal control. (**e**,**f**) Cell proliferation after transfection of miR-4435 inhibitor. (**g**,**h**) Effect of A1938 on cell proliferation. Cells were treated with A1938 (30 µM) for 36 h. All quantified data are presented as mean ± S.E.M compared to control (*p < 0.05, **p < 0.01, ***p < 0.0001). These all images are representative of 3 repeated independently repeated experiments.

We next examined whether this UQCRB-related miRNA could be regulated by a known UQCRB inhibitor, A1938^33^. After treating mutant UQCRB-expressing cell lines with A1938, levels of miR-4435 were decreased (Fig. 4a). Notably, the levels of miR-4435 were decreased in a concentration-dependent manner in CRC cells treated with A1938 (Fig. 4b). As A1938 regulated miR-4435 expression levels, we investigated the effect of A1938 on miR-4435 target, TIMP3. Consistently, the protein levels of TIMP3 were increased by A1938 treatment in the mutant UQCRB-expressing cell line (Fig. 4c) and CRC cells (Fig. 4d). These results suggest that inhibition of UQCRB by A1938 treatment is in the lineage of expressional regulation of miR-4435 and TIMP3.

In addition, we observed how cell proliferation was affected after the recovery of TIMP3 expression using miR-4435 inhibitor and A1938 treatment. First, the mutant UQCRB-expressing cells and CRC cells were treated with miR-4435 inhibitor and A1938. MiR-4435 inhibitor treatment at 20 nM and 50 nM inhibited mutant UQCRB-expressing cell proliferation (Fig. 4e). In addition, colon cancer cell proliferation was also inhibited by miR-4435 inhibitor treatment (Fig. 4f). When the cells were treated with the UQCRB inhibitor A1938, proliferation was also inhibited in a dose-dependent manner in the mutant UQCRB-expressing cells (Fig. 4g) and colon cancer cells (Fig. 4h). These results demonstrate that restoring TIMP3 expression level by A1938 and miR-4435 inhibitor treatment affects cell proliferation.

### MiR-4435 regulates migration and invasion in mutant cells and colorectal cancer cell

**Figure 5:**
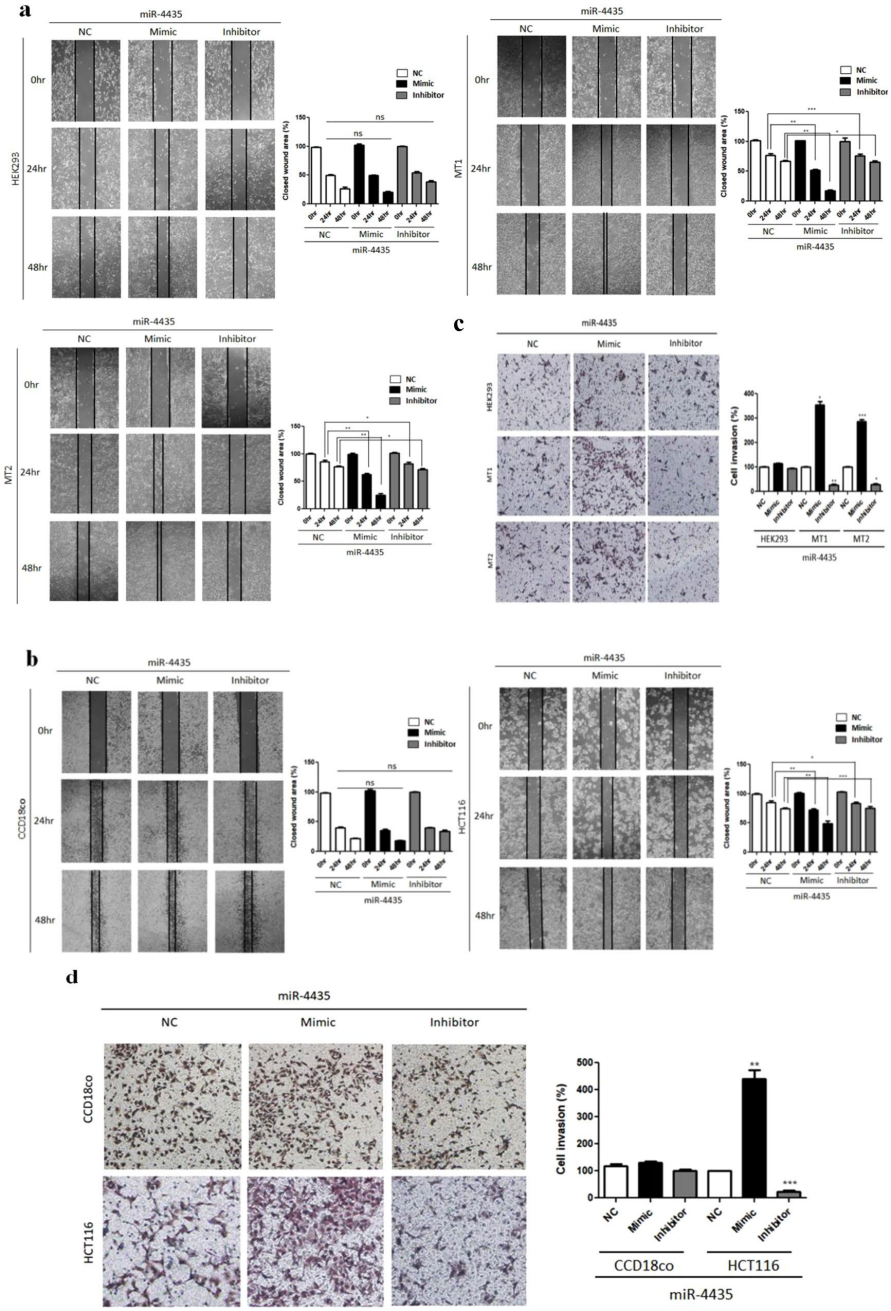
The effect of miR-4435 on migration and invasion. (**a**) HEK293, UQCRB mutant cells and (**b**) CCD18co, HCT116 were transfected with miR-4435 mimic and inhibitor for 0, 24, 48 hours. The cells migrated into the gap were counted under an optical microscope. Black lines indicate the closed wound area. Invasion assay assessed the effect of miR-4435 on cell invasion ability. (**c**,**d**) All quantified data are presented as mean ± standard error (±S.E.M) compared to control (*p < 0.05, **p < 0.01, ***p < 0.0001).

Further investigation was conducted on whether miR-4435 increases the migration and invasion ability of mutant stable cells and colorectal cancer cell. First, miR-4435 mimic and inhibitor were transfected into mutant stable cells and colorectal cancer cell. In the migration assay, when miR-4435 was transfected with mimic and inhibitor, no significant result was observed in normal cells but it was affected in mutant cells and colorectal cancer cells. The distance of cell migration was calculated to quantitatively determine the migration ability of the cells (Fig. 5a,b). Invasion assay also showed the same results as the case of migration assay (Fig. 5c,d).

As a result, overexpression of miR-4435 increased migration and invasion of mutant cell lines and colorectal cancer cells. However, in normal cells, the effect of miR-4435 was found to be relatively insignificant. Taken together, these results demonstrated that miR-4435 specifically increased the migration and invasion of the mutant stable cells and colorectal cancer cells as well.

## Discussion

MicroRNAs are a class of small non-coding RNAs that regulate various biological process by translational repression. These miRNAs are stable in blood and the expression levels of miRNAs can be stably detected from patients. These properties of miRNAs have been highlighted as suitable biomarkers for specific diseases and is an active area of research^34^. Therefore, we conducted a study based on previous research that UQCRB was highly expressed in CRC^26^. This study aimed to discover circulating miRNAs in CRC, which has previously been established to be related to abnormal UQCRB expression, and to provide insight into new biological roles of UQCRB in CRC. Furthermore, we identified a potential target mRNA of one of the circulating miRNAs and demonstrated how to regulate their levels by using a small molecule inhibitor of UQCRB. We found miR-4435 to be proportionally up-regulated with mutant UQCRB expression. After isolating exosomes from the CCM of mutant UQCRB-expressing cells, we observed increased expression levels of miR-4435 by qRT-PCR. This increased expression is supported by increased miR-4435 expression levels in human serum sample-derived exosomes. Further, increase in miR-4435 expression levels is strongly correlated with stage progression of CRC. In parallel, we used mRNA-sequencing of the mutant UQCRB-expressing cell line and miRDB analysis for miRNA target prediction to elucidate likely target candidates of miR-4435. We identified the tumor suppressor gene, *TIMP3*, and found that its protein expression was downregulated in mutant UQCRB-expressing cell lines and CRC cells. It is known that the tumor inhibitor of metalloproteinase-3 (TIMP3) promoter is hypermethylated in approximately 30% of CRC which is predicted to silence the expression of TIMP3^35^. Furthermore both mRNA and protein levels of tissue inhibitor of metalloproteinase-3 (TIMP3) were decreased significantly in colorectal cancer tissue when compared with normal mucosa, suggesting that decrease of TIMP3 expression is correlated with malignant behavior of colorectal cancer^32^. The relationship between miR-4435 and *TIMP3* was verified by a dual luciferase reporter assay using a psiCHECK-2 vector that contains TIMP3 3′-UTR segment with wild-type or mutated miRNA seed sequence. In addition, inhibition of miR-4435 also confirmed that the protein expression level of TIMP3 was increased. Furthermore, we verified that the expression levels of miR-4435 and *TIMP3* were regulated by the UQCRB inhibitor, A1938. Taken together, it was concluded that miR-4435 increased migration, invasion of mutant stable cells and colorectal cancer cell. In summary, we identified a key-miRNA and mRNA related to UQCRB and new biological roles of UQCRB in UQCRB-related cancer, particularly CRC. This study also demonstrated the potential of miR-4435 for diagnosis of UQCRB-related cancer of CRC. In addition, we confirmed that *TIMP3*, the target of miR-4435, is regulated by the UQCRB inhibitor, A1938. As a therapeutic, miRNAs can block only the target proteins by targeting their mRNAs, thus regulating the expression level of the relevant proteins without side effects. To this end, studies are underway to deliver miRNAs *in vivo* through chemical synthesis of miRNA^36^. Small molecules targeting UQCRB, such as A1938, has also a therapeutic value to treat CRC particularly overexpressing UQCRB and miR-4435 as well for precision oncology treatment. Following studies on the biological processes underlying the increase in miR-4435 levels in mutant UQCRB-expressing cell lines in transcriptional levels and correlation of miR-4435 with the progression and metastasis of CRC *in vivo* will help to understand the biological and clinical values of UQCRB-miR-4435-TIMP3-CRC links.

## Materials and Methods

All methods were performed in accordance with the relevant guidelines and regulations.

### Cell culture

Control (HEK293, human normal kidney cells) and mutant UQCRB-expressing cell lines, MT1 and MT2 in HEK293 cells, were grown in Dulbecco’s modified Eagle’s medium (DMEM; Invitrogen, Grand Island, NY), supplemented with 10% fetal bovine serum (FBS; Invitrogen) and 1% antibiotics (Invitrogen). HCT116 (human colon cancer cells) was grown in RPMI1640 (Invitrogen), with the same condition as described above. Human normal colon cells, CCD18Co, were grown in Dulbecco’s modified Eagle’s medium supplemented with 20% fetal bovine serum (Gibco BRL) and 1× non-essential amino acids (Sigma-Aldrich, St. Louis, MO, USA). All cells were incubated in media (pH 7.4) at 37 °C in a humidified incubator with 5% CO_2_. To maintain the mutant stable cell lines, 1 mg/mL G418 was applied in the DMEM media.

### RNA isolation

Cells were collected with Trizol (Invitrogen, Carlsbad, CA) and total RNA was extracted using PureLink^TM^ RNA isolation kit (Ambion) according to the manufacturer’s instructions. RNA isolation from exosomes was conducted by SeraMir^TM^ (System Biosciences) according to the manufacturer’s instructions.

### miRNA sequencing and miRNA expression

Isolated total RNAs were applied to miRNA-sequencing (Illumina HiSeq 2000) and the raw data set was acquired from Macrogen (Macrogen Inc., Seoul, South Korea). miRNA sequencing data are available in the GEO repository under accession number SAMN13680025 (www.ncbi.nlm.nih.gov/geo/). In brief, for miRNA-sequencing, small RNA sample preparation was done according to Illumina’s protocol. 5′ and 3′ adapters were sequentially ligated to small RNAs of 18–30 bases that were gel purified from 5–10 µg total RNA. Adapter-ligated small RNAs were trimmed, reverse transcribed, amplified, and sequenced on a HiSeq 2000 (Illumina) following manufacturer’s instructions. Next, adapters were trimmed and mapped to a reference genome using Bowtie tools^37^. Using edgeR, the data were normalized by quartile method. Finally, we normalized reads to counts per million (CPM) and calculated fold change (FC). A false discover rate (FDR) was controlled by adjusting *p*-value using the Benjamini-Hochberg algorithm to identify candidate miRNAs. The transcriptome analysis data of mutant UQCRB-expressing cells are available at Korean BioInformation Center (KBRS20171018–0000001~KBRS20171018–0000336).

### mRNA sequencing

We performed mRNA-sequencing (Illumina HiSeq 2000) on total RNA and the raw data set was acquired from Macrogen (Macrogen Inc., Seoul, South Korea). In brief, we used 1 µg of total RNA to construct cDNA libraries with the TruSeq RNA library kit. The protocol consisted of polyA-selected RNA extraction, RNA fragmentation, random hexamer primed reverse transcription, and 100 nt paired-end sequencing by Illumina HiSeq 2000. The libraries were quantified using qRT-PCR according to the qRT-PCR quantification protocol guide and quantified using an Agilent Technologies 2100 Bioanalyzer. Next, adapters of these mRNA reads were trimmed and mapped to a reference genome using TopHat2. The reference genome sequence (hg19, Genome Reference Consortium GPCh37) and annotation data were downloaded from the UCSC website (http://genome.ucsc.edu).

### Quantitative RT-PCR (qRT-PCR)

Isolated RNAs were reverse-transcribed with TaqMan miRNA reverse transcription kit (Applied Biosystems, Waltham, MA) and Taqman primers (Applied Biosystems). Real-time PCR was performed on a HT Fast Real Time PCR system (Applied Biosystems) with Taqman Fast Universal PCR master mix (Applied Biosystems) or LightCycler 96 system (Roche) with FastStart Essential DNA Probes Master (Roche) and TaqMan primers (Applied Biosystems) according to the manufacturer’s instructions. Data were calculated by the 2-^ΔΔCT^ method and normalized to the internal control small RNA, *RNU48*.

### Western blot analysis

Cell lysates were analyzed via 12.5% sodium dodecyl sulfate polyacrylamide gel electrophoresis (SDS-PAGE) and subsequently transferred to polyvinylidene difluoride membranes (Millipore, Billerica, MA) following standard methods. Blots were incubated at 4 °C overnight with the following primary antibodies: anti-UQCRB (1:500) (A78559, Sigma-Aldrich, Saint Louis, MO), anti-TIMP3 (1:3000) (ab39184, Abcam, Cambridge, MA), and β-actin (1:3000) (ab6276, Abcam, Cambridge, MA). Immunolabeling was performed using Clarity Western ECL substrate (Bio-Rad, Hercules, CA). Images were quantified using Image LabTM software (Bio-Rad).

### Exosome isolation

Exosomes were extracted from cell culture media (CCM) with ExoQuick-TC^TM^. Cells (3 × 10^5^) were seeded onto 100 mm cell culture plates for 72 h and incubated at 37 °C in a humidified incubator with 5% CO_2_, pH 7.4. After 72 h, the ExoQuick^TM^ kit was used to isolate exosome from sera of colon cancer patients according to the manufacturer’s instruction.

### Luciferase assay

A cDNA [47-bp, nt 3971–4017 (NM_000362.4)] bearing the predicted target of miR-4435 or the same cDNA with mismatched target sequences (the 7-nt core seed sequence, CTGGCCA, was replaced with GACCGCT) was inserted into XhoI and NotI sites in psiCHECK-2 vector (Promega, Madison, WI, USA) to construct psiCHECK-2_TIMP3(WT) or psiCHECK-2_TIMP3(MT), respectively. The inserted DNA sequences of TIM3-WT and -MT were verified by DNA sequencing (Supplementary Fig. 4). HEK293 cells were co-transfected with each reporter vector (25 ng) and miRNA mimics (50 nM) using Lipofectamine 2000 (Invitrogen). miR-10a-5p mimic (5′-UACCCUGUAGAUCCGAAUUUGUG-3′) used as a control was purchased from Ambion (Thermo Fisher Scientific, Waltham, MA, USA). *Renilla* luciferase (Rluc) and firefly luciferase (Fluc) activities were measured 24 h after transfection using the Dual-Glo luciferase assay system (Promega).

### miRNA inhibitor transfection

Mutant UQCRB-expressing cells and colon cancer cells were transfected with miR-4435 inhibitor purchased from Bioneer Co. (Daejeon, Korea). Cells (1.5 × 10^5^) were seeded in six-well plates 48 h prior to transfection. Transfection was performed using Lipofectamine RNAiMAX (Invitrogen) reagent according to the manufacturer’s instruction.

### Cell proliferation assay

Cells (3 × 10^3^) were seeded in 96-well plates and incubated for 24–72 h. Cell proliferation was measured using 3-(4,5-dimethylthiazol-2-yl)-2,5-diphenyltetrazolium bromide (MTT; Sigma-Aldrich, Saint Louis, MO) colorimetric assay. Cells were treated with UQCRB inhibitor (A1938) and transfected with miR-4435 inhibitor.

### Migration assay

Cell migration ability was evaluated using a wound healing assay. Cells were seeded in 6-well plates and transfected with miR-4435 mimic and inhibitor. Then, cells allowed to reach 80% confluence. A wound was artificially created by scratching the cell monolayer with a 200 μl pipette tip. Wound closure was observed at 0 and 24, 48 hours, and the image was photographed using a microscope. The migration distance (MD) in each group was calculated according to the Image J.

### Invasion assay

Cell invasion was assayed using a Transwell® chamber system with polycarbonate filter inserts with a pore size of 8.0 µm (Corning Costar, Acton, MA, USA). The upper side was coated with 10 µl Matrigel (3 mg/ml). The cells (7 × 104) transfected with miR-4435 mimic and inhibitor, were collected serum-free media and placed in the upper chamber of the filter. Then, the lower chamber filled with FBS containing media. The chamber was incubated at 37 °C for 16 h, and the cells were subsequently fixed with methanol and stained with hematoxylin/eosin. The total number of cells that invaded the lower chamber of the filter was counted using an optical microscope (Olympus).

### Statistical analysis

Results are expressed as mean ± standard error (±S.E.M.) and all statistical analyses were calculated with GraphPad Prism (ver. 5.00 for Windows, GraphPad Software, San Diego, CA, www.graphpad.com). Student’s *t*-test was used to determine statistical significance between control and test groups. A *p*-value less than 0.05 was considered statistically significant (* indicates *p* < 0.05, ** indicates *p* < 0.01, *** indicates *p* < 0.001).

### Ethics statement

Serum samples of colorectal cancer patients were obtained from the Biobank, Severance Hospital, Yonsei University College of Medicine, Seoul, Korea (IRB Approval No. 7001988-201803-BR-141-01E), and written informed consent was obtained from all patients.

## Supplementary information

Supplementary information.

## References

[1] Nunnari J, Suomalainen A (2012). Mitochondria: in sickness and in health. Cell..

[2] Wallace DC (2012). Mitochondria and cancer. Nat Rev Cancer..

[3] Dromparis P, Michelakis ED (2013). Mitochondria in vascular health and disease. Annu Rev Physiol..

[4] Petersen KF, Dufour S, Befroy D, Garcia R, Shulman GI (2004). Impaired mitochondrial activity in the insulin-resistant offspring of patients with type 2 diabetes. N Engl J Med..

[5] Friedman JR, Nunnari J (2014). Mitochondrial form and function. Nature..

[6] Suzuki H, Hosokawa Y, Toda H, Nishikimi M, Ozawa T (1988). Cloning and sequencing of a cDNA for human mitochondrial ubiquinone-binding protein of complex III. Biochem Biophys Res Commun..

[7] Jung HJ, Kwon HJ (2013). Exploring the role of mitochondrial UQCRB in angiogenesis using small molecules. Mol Biosyst..

[8] Jung HJ (2013). Mitochondrial UQCRB regulates VEGFR2 signaling in endothelial cells. J Mol Med (Berl)..

[9] Jung HJ (2010). Terpestacin inhibits tumor angiogenesis by targeting UQCRB of mitochondrial complex III and suppressing hypoxia-induced reactive oxygen species production and cellular oxygen sensing. J Biol Chem..

[10] Jia HL (2007). Gene expression profiling reveals potential biomarkers of human hepatocellular carcinoma. Clin Cancer Res..

[11] Wrzeszczynski KO (2011). Identification of tumor suppressors and oncogenes from genomic and epigenetic features in ovarian cancer. PLoS One..

[12] Harada T, Chelala C, Crnogorac-Jurcevic T, Lemoine NR (2009). Genome-wide analysis of pancreatic cancer using microarray-based techniques. Pancreatology..

[13] Lascorz J (2012). Polymorphisms in the mitochondrial oxidative phosphorylation chain genes as prognostic markers for colorectal cancer. BMC Med Genet..

[14] Haut S (2003). A deletion in the human QP-C gene causes a complex III deficiency resulting in hypoglycemia and lactic acidosis. Hum Genet..

[15] Chang J (2014). A mutation in the mitochondrial protein UQCRB promotes angiogenesis through the generation of mitochondrial reactive oxygen species. Biochem Biophys Res Commun..

[16] Bartel DP (2009). MicroRNAs: target recognition and regulatory functions. Cell.

[17] Esquela-Kerscher A, Slack FJ (2006). Oncomirs - microRNAs with a role in cancer. Nat Rev Cancer..

[18] Kent OA, Mendell JT (2006). A small piece in the cancer puzzle: microRNAs as tumor suppressors and oncogenes. Oncogene..

[19] Ambros V (2004). The functions of animal microRNAs. Nature..

[20] Bartel DP (2004). MicroRNAs: genomics, biogenesis, mechanism, and function. Cell..

[21] Kim, J. E. *et al*. Hsa-miR-10a-5p downregulation in mutant UQCRB-expressing cells promotes the cholesterol biosynthesis pathway. Sci Rep. **1**, 10.1038/s41598-018-30530-6 (2018). 10.1038/s41598-018-30530-6 PMC6098055 30120311

[22] Chim SS (2008). Detection and characterization of placental microRNAs in maternal plasma. Clin Chem..

[23] Hu Z (2010). Serum microRNA signatures identified in a genome-wide serum microRNA expression profiling predict survival of non-small-cell lung cancer. J Clin Oncol..

[24] Lv ZC, Fan YS, Chen HB, Zhao DW (2015). Investigation of microRNA-155 as a serum diagnostic and prognostic biomarker for colorectal cancer. Tumour biology: the journal of the International Society for Oncodevelopmental Biology and Medicine..

[25] De Rosa M (2015). Genetics, diagnosis and management of colorectal cancer. Oncol Rep..

[26] Kim HC (2017). Mitochondrial UQCRB as a new molecular prognostic biomarker of human colorectal cancer. Exp Mol Med..

[27] Wang X (2016). Screening miRNAs for early diagnosis of colorectal cancer by small RNA deep sequencing and evaluation in a Chinese patient population. Onco Targets Ther..

[28] Slattery Martha L, Herrick Jennifer S, Mullany Lila E, Wolff Erica, Hoffman Michael D, Pellatt Daniel F, Stevens John R, Wolff Roger K (2016). Colorectal tumor molecular phenotype and miRNA: expression profiles and prognosis. Modern Pathology.

[29] Liu Jixi, Liu Fang, Li Xiaoou, Song Xin, Zhou Lei, Jie Jianzheng (2017). Screening key genes and miRNAs in early-stage colon adenocarcinoma by RNA-sequencing. Tumor Biology.

[30] Cha DJ (2015). KRAS-dependent sorting of miRNA to exosomes. elife..

[31] Slaby O (2007). Altered expression of miR-21, miR-31, miR-143, and miR-145 is related to clinicopathologic features of colorectal cancer. Oncology..

[32] Lin H (2012). Tissue inhibitor of metalloproteinases-3 transfer suppresses malignant behaviors of colorectal cancer cells. Cancer Gene Ther..

[33] Jung HJ, Cho M, Kim Y, Han G, Kwon HJ (2014). Development of a novel class of mitochondrial ubiquinol-cytochrome c reductase binding protein (UQCRB) modulators as promising antiangiogenic leads. J Med Chem..

[34] Wang H (2018). Circulating microRNAs as potential cancer biomarkers: the advantage and disadvantage. Clin Epigenetics..

[35] Lao JVV (2012). The Role of Timp3 in the Pathogenesis of Colorectal Cancer and Timp3 Promoter Methylation as a Potential Predictive Marker for Egfr Inhibitor Therapy. Surg Res..

[36] Almanza G (2013). Synthesis and delivery of short, noncoding RNA by B lymphocytes. Proc Natl Acad Sci USA.

[37] Langmead, B. Aligning short sequencing reads with Bowtie. Curr Protoc Bioinformatics Chapter 11, Unit 11 17, 10.1002/0471250953.bi1107s32 (2010). 10.1002/0471250953.bi1107s32 PMC3010897 21154709

