# Supplementary material for: MiR-4435 is an UQCRB-related circulating miRNA in human colorectal cancer
Source: Sci Rep. 2020 Feb 18;10:2833. doi: 10.1038/s41598-020-59610-2 (PMC7029028; doi:10.1038/s41598-020-59610-2)

# Supplementary data

**MiR-4435 is an UQCRB-related circulating miRNA in human colorectal cancer**

Ji Won Hong^1,§^, Jung Min Kim^1,§^, Jeong Eun Kim^1^, Hee Cho^1^, Dasol Kim^1^,

Wankyu Kim^2^, Jong-Won Oh^1^, and Ho Jeong Kwon^1,3, *^

^1^Department of Biotechnology, College of Life Science and Biotechnology, Yonsei University, Seoul 03722, Republic of Korea
^2^Ewha Research Center for Systems Biology, Division of Molecular & Life Sciences, Ewha Womans University, Seoul, Republic of Korea
^3^Department of Internal Medicine, Yonsei University College of Medicine, Seoul 03722, Republic of Korea.

Running title: MiR-4435 as a circulating miRNA biomarker of human colorectal cancer

To whom correspondence should be addressed: Ho Jeong Kwon, Chemical Genomics Global Research Laboratory, Department of Biotechnology, College of Life Science & Biotechnology, Yonsei University, Seoul 120-749, Republic of Korea, Tel:82-2-2123-5883; Fax:82-2-362-7265; E-mail: [kwonhj@yonsei.ac.kr](mailto:kwonhj@yonsei.ac.kr)

All methods were performed in accordance with the relevant guidelines and regulations.

**Supplementary Table 1** Summary of the number of mRNAs and miRNAs, and the statistical results for differential gene expression analysis

<mRNA-sequencing data>

| **Gene** | **Description** | **HEK293 mean** | **MT1**  **mean** | **MT1**  **p-value** | **MT2**  **mean** | **MT2**  **p-value** |
| --- | --- | --- | --- | --- | --- | --- |
| TIMP3 | TIMP metallopeptidase inhibitor 3 | 3.27319509 | 2.132797397 | 0.132129755 | 2.978213267 | 0.079176453 |
| TLE2 | transducin-like enhancer of split 2 (E(sp1) homolog, Drosophila) | 2.415907343 | 1.056263557 | 0.270468542 | 1.923267993 | 0.40745 |
| RBPMS2 | RNA binding protein with multiple splicing 2 | 3.583661127 | 2.22864259 | 0.087371474 | 3.217546807 | 0.336854054 |
| DAAM2 | dishevelled associated activator of morphogenesis 2 | 3.00447113 | 1.84096394 | 0.910185709 | 2.572301977 | 0.578801268 |
| CORO2A | coronin, actin binding protein, 2A | 2.038293393 | 0.865248663 | 0.151943844 | 1.630379637 | 0.061370028 |
| NID2 | nidogen 2 (osteonidogen) | 2.885456073 | 1.73951689 | 0.076661941 | 2.437260913 | 0.112992885 |
| UNC5B | unc-5 homolog B  (C. elegans) | 4.00949789 | 2.907769783 | 0.123587738 | 3.347202987 | 0.482126166 |

<miR-sequencing data>

| **microRNA** | **HEK293_1** | **HEK293_2** | **UQCRB**  **Mutant1_1** | **UQCRB**  **Mutant1_2** | **log FC** | **Log CPM** | **P-Value** | **FDR** |
| --- | --- | --- | --- | --- | --- | --- | --- | --- |
| hsa-miR-4485 | 29 | 26 | 63 | 204 | 2.4145 | 2.5680 | 2.40.E-05 | 6.54.E-04 |
| hsa-miR-4745-5p | 67 | 61 | 265 | 268 | 2.0065 | 3.4065 | 2.10.E-04 | 4.92.E-03 |
| hsa-miR-1908-3p | 163 | 208 | 437 | 838 | 1.7862 | 4.7350 | 6.55.E-04 | 1.30.E-02 |
| hsa-miR-1226-3p | 100 | 95 | 231 | 202 | 1.1541 | 3.3177 | 2.74.E-02 | 1.99.E-01 |
| hsa-miR-4435 | 93 | 73 | 392 | 212 | 1.7962 | 3.6088 | 7.28.E-0 | 1.41.E-02 |
| hsa-miR-21-3p | 1,725 | 2,301 | 3,103 | 4,053 | 1.6689 | 8.4240 | 1.18.E-03 | 2.12.E-02 |
| **microRNA** | **HEK293_1** | **HEK293_2** | **UQCRB**  **Mutant2_1** | **UQCRB**  **Mutant2_2** | **log FC** | **Log CPM** | **P-Value** | **FDR** |
| hsa-miR-4485 | 21 | 21 | 71 | 114 | 2.1167 | 2.1465 | 9.63.E-06 | 4.11.E-04 |
| hsa-miR-4745-5p | 50 | 49 | 88 | 32 | 0.2659 | 2.0764 | 5.63.E-01 | 9.51.E-01 |
| hsa-miR-1908-3p | 122 | 168 | 360 | 387 | 1.3707 | 4.2870 | 1.08.E-03 | 2.57.E-02 |
| hsa-miR-1226-3p | 116 | 91 | 120 | 228 | 0.7457 | 3.3988 | 7.65.E-02 | 4.87.E-01 |
| hsa-miR-4435 | 70 | 59 | 97 | 92 | 0.5500 | 2.6088 | 2.07.E-01 | 7.24.E-01 |
| hsa-miR-21-3p | 1,725 | 2,301 | 3,103 | 4,053 | 0.8301 | 7.7068 | 3.86.E-02 | 3.33.E-01 |


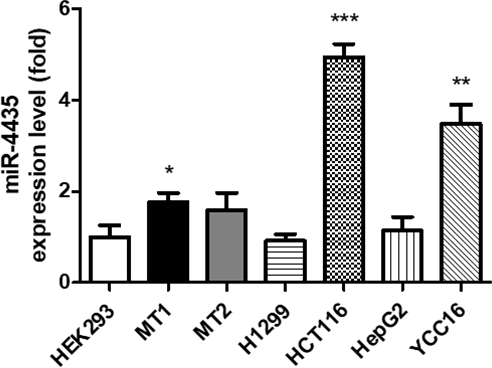


**Supplementary Figure 1** miR-4435 was up-regulated in colorectal cancer cell lines. Validation of selected miRNAs in various cancer cell line using qRT-PCR. Expression levels of candidate miR-4435 in gastric cancer (YCC16), liver cancer (HepG2), and lung cancer (H1299) and colorectal cancer cells (HCT116). All quantified data were presented as mean ± standard error (± S.E.M) compared to control (*p < 0.05, **p < 0.01, ***p < 0.0001).


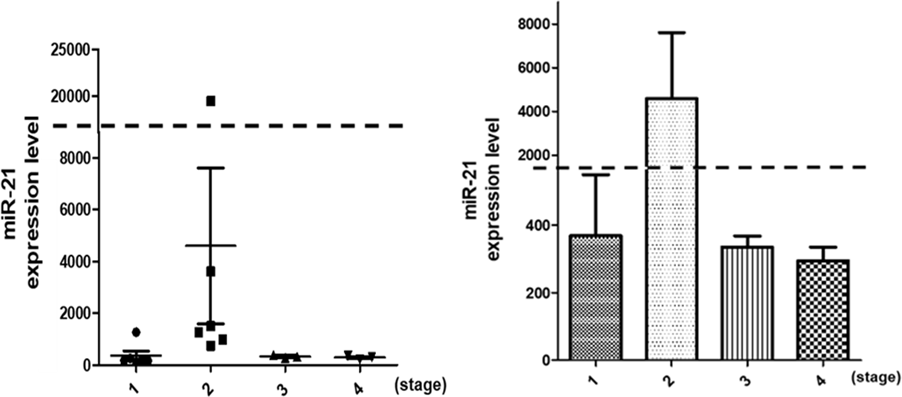


**Supplementary Figure 2** Expression levels of miR-21 in serum of colorectal cancer patients using qRT-PCR. The expression level of each patients (left), and the mean expression level of miR-21 in each stage (right). All quantified data are presented as mean ± S.E.M compared to control (*p < 0.05, **p < 0.01, ***p < 0.0001).

TIMP3_MT1

GGCTCCACTTCAGCCAGGAGGACGCTCCAGATGAAATGGGTAAGTACATCAAGAGCTTCGTGGAGCGCGTGCTGAAGAACGAGCAGTAATTCTAGGCGATCGCTCGAGGTGGACATCAGTGTCTTCTCTGTGAGGCATGACCGCTTTCGCACTCCGCGGCCGCTGGCCGCAATAAAATATCTTTATTTTCATTACATCTGTGTGTTGGTTTTTTGTGTGAGGATCTAAATGAGTCTTCGGACCTCGCGGGGGCCGCTTAAGCGGTGGTTAGGGTTTGTCTGACGCGGGGGGAGGGGGAAGGAACGAAACACTCTCATTCGGAGGCGGCTCGGGGTTTGGTCTTGGTGGCCACGGGCACGCAGAAGAGCGCCGCGATCCTCTTAAGCACCCCCCCGCCCTCCGTGGAGGCGGGGGTTTGGTCGGCGGGTGGTAACTGGCGGGCCGCTGACTCGGGCGGGTCGCGCGCCCCAGAGTGTGACCTTTTCGGTCTGCTCGCAGACCCCCGGGCGGCGCCGCCGCGGCGGCGACGGGCTCGCTGGGTCCTAGGCTCCATGGGGACCGTATACGTGGACAGGCTCTGGAGCATCCGCACGACTGCGGTGATATTACCGGAGACCTTCTGCGGGACGAGCCGGGTCACGCGGCTGACGCGGAGCGTCCGTTGGGCGACAAACACCAGGACGGGGCACAGGTACACTATCTTGTCACCCGGAGGCGCGAGGGACTGCAGGAGCTTCAGGGAGTGGCGCAGCTGCTTCATCCCCGTGGCCCGTTGCTCGCGTTTGCTGGCGGTGTCCCCGGAAGAAATATATTTGCATGTCTTTAGTTCTATGATGACACAAACCCCGCCCAGCGTCTTGTCATTGGCGAATTCGAACACGCAGATGCAGTCGGGGCGGCGCGGTCCCAGGTCCACTTCGCATATTAAGGTGACGCGTGTGGCCTCGAACACCGAGCGACCCTGCAGCGACCCGCTTAAAAGCTTGGCATTCCGGTACTGTTGGTAAAGCCACCATGGCC

TIMP3_WT

GGCTCCACTTCAGCCAGGAGGACGCTCCAGATGAAATGGGTAAGTACATCAAGAGCTTCGTGGAGCGCGTGCTGAAGAACGAGCAGTAATTCTAGGCGATCGCTCGAGGTGGACATCAGTGTCTTCTCTGTGAGGCATCTGGCCATTCGCACTCCGCGGCCGCTGGCCGCAATAAAATATCTTTATTTTCATTACATCTGTGTGTTGGTTTTTTGTGTGAGGATCTAAATGAGTCTTCGGACCTCGCGGGGGCCGCTTAAGCGGTGGTTAGGGTTTGTCTGACGCGGGGGGAGGGGGAAGGAACGAAACACTCTCATTCGGAGGCGGCTCGGGGTTTGGTCTTGGTGGCCACGGGCACGCAGAAGAGCGCCGCGATCCTCTTAAGCACCCCCCCGCCCTCCGTGGAGGCGGGGGTTTGGTCGGCGGGTGGTAACTGGCGGGCCGCTGACTCGGGCGGGTCGCGCGCCCCAGAGTGTGACCTTTTCGGTCTGCTCGCAGACCCCCGGGCGGCGCCGCCGCGGCGGCGACGGGCTCGCTGGGTCCTAGGCTCCATGGGGACCGTATACGTGGACAGGCTCTGGAGCATCCGCACGACTGCGGTGATATTACCGGAGACCTTCTGCGGGACGAGCCGGGTCACGCGGCTGACGCGGAGCGTCCGTTGGGCGACAAACACCAGGACGGGGCACAGGTACACTATCTTGTCACCCGGAGGCGCGAGGGACTGCAGGAGCTTCAGGGAGTGGCGCAGCTGCTTCATCCCCGTGGCCCGTTGCTCGCGTTTGCTGGCGGTGTCCCCGGAAGAAATATATTTGCATGTCTTTAGTTCTATGATGACACAAACCCCGCCCAGCGTCTTGTCATTGGCGAATTCGAACACGCAGATGCAGTCGGGGCGGCGCGGTCCCAGGTCCACTTCGCATATTAAGGTGACGCGTGTGGCCTCGAACACCGAGCGACCCTGCAGCGACCCGCTTAAAAGCTTGGCATTCCGGTACTGTTGGTAAAGCCACCATGGCC

**Supplementary Figure 3** Results of DNA Sequences of TIM3-WT and MT. TIMP3 3'-UTR segments of WT and MT are colored in red.

**Full Western Blot Images from Figures**

Supp. Fig. 2b Supp. Fig. 2d


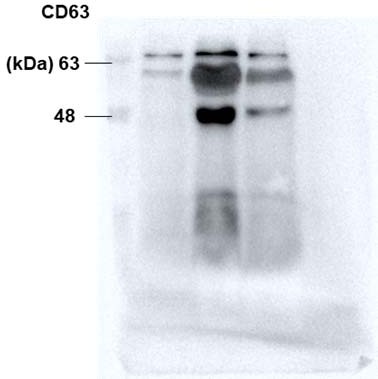

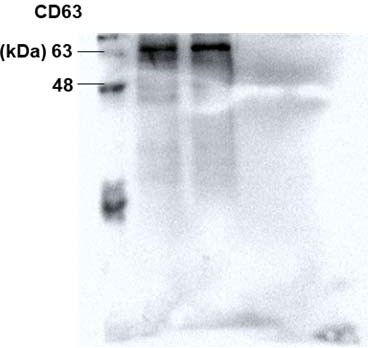


Supp. Fig. 3b Supp. Fig. 3b


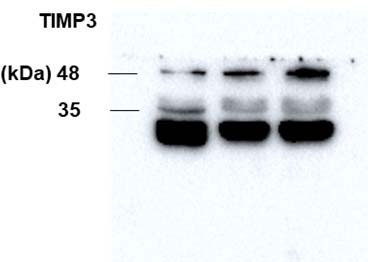

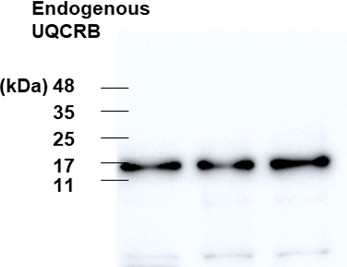


Supp. Fig. 3b Supp. Fig. 3c


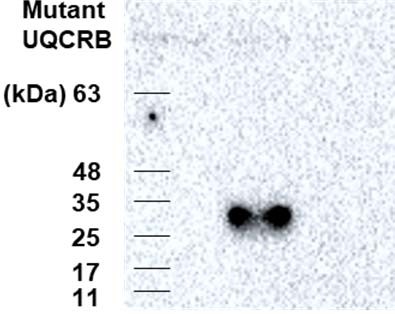

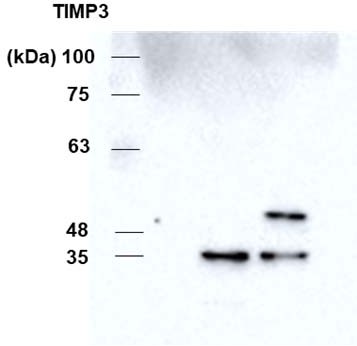


Supp. Fig. 3c Supp. Fig. 3f


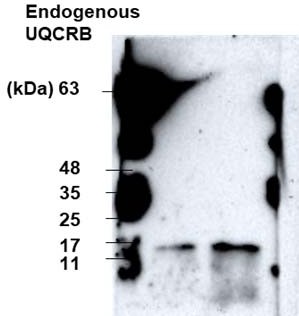

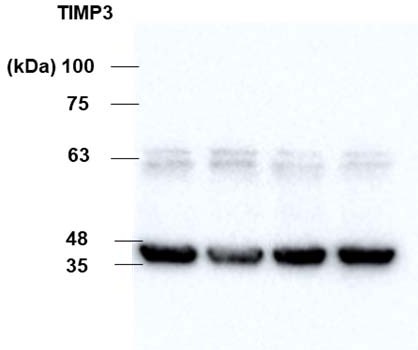


Supp. Fig. 3g Supp. Fig. 4c


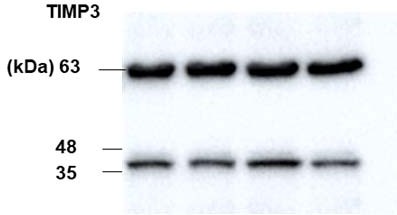

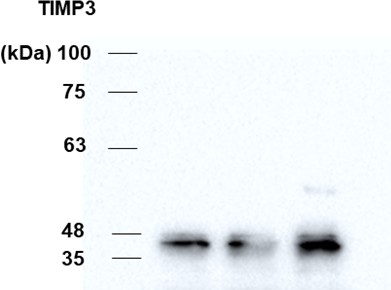


Supp. Fig. 4d


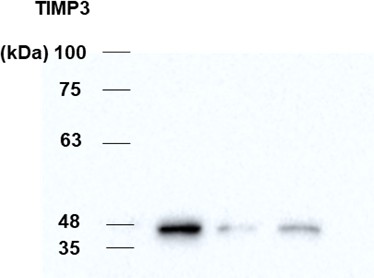

Supplement: Supplementary file 1 — Supplementary information. [file 41598_2020_59610_MOESM1_ESM.docx]
